# Construction Notes

**Published:** 1907-07-06

**Authors:** 


### CONSTRUCTION NOTES.

Bognor Urban Council has resolved to spend a sum of £500 to complete the local isolation hospital.

It is proposed to erect a new convalescent home in connection with the Montrose Asylum and Infirmary at Edzell at an estimated cost of £2,000.

A sum of £5,000 is required for the proposed new laboratories in connection with the Glasgow Cancer Hospital. Only some £1,280 has so far been received.

The work on the new Manchester Royal Infirmary is making good progress. The out-patient block will be started this month, together with the pathological and septic blocks.

It is proposed to extend and improve the extern department of the Belfast Ophthalmic Institution and Eye and Ear Hospital, which is at present badly ventilated. The suggested alterations are estimated to cost £300.

An operating theatre, on the latest pattern and thoroughly fitted up, has been added to the West Cornwall Miners' Hospital at Redruth. The theatre, which is tiled in marble, cost £1,500, and attached to it are anæsthetic and sterilising rooms.

The Committee of the Newcastle Dental Hospital has arranged for a seven years' lease of the present premises, into which the hospital moved in October last. A sum of £300 has been expended on improvements connected with the ventilating and heating systems.

In the annual report of the Glasgow Children's Hospital, the Governors advise against any extension of the institution on its present site. They recommend the building of a large children's hospital to contain at least 250 cots on a new site in the city, and propose to effect certain necessary extensions of the country branch at Drumchapel. The estimates for the new building and proposed extension is £100,000.

